# A Systematic Review of Postoperative Pain Outcome Measurements Utilised in Regional Anesthesia Randomized Controlled Trials

**DOI:** 10.1155/2018/9050239

**Published:** 2018-07-29

**Authors:** E. Pushpanathan, T. Setty, B. Carvalho, P. Sultan

**Affiliations:** ^1^Department of Anaesthesia, Guy's and St. Thomas' NHS Foundation Trust, London, UK; ^2^Department of Anaesthesia, University College Hospitals London NHS Foundation Trust, London, UK; ^3^Department of Anesthesia, Stanford University School of Medicine, Stanford, CA, USA; ^4^Department of Anaesthesia, University College Hospitals London NHS Foundation Trust, University College London, London, UK

## Abstract

**Introduction:**

Regional anesthesia is a rapidly growing subspecialty. There are few published meta-analyses exploring pain outcome measures utilised in regional anesthesia randomized controlled trials (RCTs), which may be due to heterogeneity in outcomes assessed. This systematic review explores postoperative pain outcomes utilised in regional anesthesia RCTs.

**Methods:**

A literature search was performed using three databases (Medline, Embase, and CINAHL). Regional anesthesia RCTs with postoperative pain as a primary outcome were included if written in English and published in one of the top 20 impact factor journals between 2005 and 2017. Study quality was assessed using the Cochrane Collaboration's tool for assessing risk of bias.

**Results:**

From the 31 included articles, 15 different outcome measures in total were used to assess postoperative pain. The most commonly (16/31) used outcome measures were verbal numerical grading of pain out of 10, total opioid consumption, and visual analogue scale 10 cm (VAS). The need for analgesia was used as an outcome measure where studies did not use a pain rating score. Ten studies reported pain scores on activity and 27/31 studies utilised ≥2 pain outcomes. Time of measurement of pain score also varied with a total of 51 different time points used in total.

**Conclusion:**

Analysis of the articles demonstrated heterogeneity and inconsistency in choice of pain outcome and time of measurement within regional anesthesia studies. Identification of these pain outcomes utilised can help to create a definitive list of core outcomes, which may guide future researchers when designing such studies.

## 1. Introduction

Regional anesthesia is a rapidly growing subspecialty, with a widening spectrum of applications and uses. Despite growth in this area of research, there have been few published regional anesthesia systematic reviews, meta-analyses, Cochrane reviews, or National Institute for Health and Care Excellence (NICE) guidelines exploring pain outcomes. This may be due to the heterogeneity of outcome variables chosen in regional anesthesia studies, making it difficult to combine and analyse data.

The Cochrane Collaboration, which aims to give the “clinical bottom line” through its reviews, has 39 reviews, which mention regional anesthesia. These reviews commonly cite the outcomes chosen as “incomplete,” “poor quality,” and “heterogeneous,” which impeded the authors' ability to draw meaningful conclusions [1–4]. Additionally, there are four NICE guidelines centered on regional anesthesia [5–8], and of these, only one deals specifically with the use of regional anesthesia to manage surgical or postoperative pain [5].

Identification of outcomes utilised can subsequently help to create a definitive list of core outcomes, which may guide future researchers when designing studies. This systematic review aimed to explore outcomes utilised in regional anesthesia randomized controlled trials (RCTs) to measure postoperative pain.

## 2. Methods

We have adhered to the Preferred Reporting Items for Systematic Reviews and Meta-Analyses (PRISMA) statement standards in this article [9].

We performed a literature search using three search engines (Medline, Embase, and CINAHL). These databases were searched for RCTs published between 2005 and 2017. The search strategy included manual searching of citations for further relevant articles. The search was initially performed in mid December 2016 and repeated on January 5th 2017. An example of the exact search terms used for each database is included in Appendix A. The review was limited to published English language RCTs exploring regional anesthesia, with a primary outcome of postoperative pain. Articles were included if published between 2005 and 2017, in one of the top 20 impact factor journals (Appendix B, Table 4). Since it was felt that the assessment of pain within the adult population is significantly different to the pediatric and obstetric populations, this review was limited only to adult studies (participants aged 18 years and over).

The selected studies were analysed by two of the authors (E. Pushpanathan and T. Setty). Each study was read and the following data were extracted and tabulated: authors, year of publication, postoperative pain outcome measures utilised, times of postoperative pain assessment, nerve block studied, and personnel collecting the data. The two primary outcomes explored in this systematic review were the type of pain outcome measured and the time of measurement.

The quality of studies included in this systematic review was evaluated using the Cochrane Collaboration's tool for assessing risk of bias [10]. Areas of methodological quality assessed included concealment of allocation, random sequence generation, blinding of the assessors and participants, and accounting for all subjects. Overall quality was graded as low (low risk of bias), high (high risk of bias), or unclear risk of bias for each domain entry [10]. The quality of each study was also assessed using the Jadad score, which examines withdrawals, blinding, and randomization of a study [11], although studies were not excluded on the basis of this assessment. At least two individuals extracted the study data independently utilizing a standardised review protocol and recorded the information on a data spreadsheet. Differences were resolved by reexamination of the original manuscripts and by discussion. The data were then entered into a Microsoft Excel for Mac 2016 spreadsheet (Microsoft Corporation, Redmond, WA) by one of the authors (E. Pushpanathan) and checked by a second investigator (T. Setty).

## 3. Results

**Figure 1:**
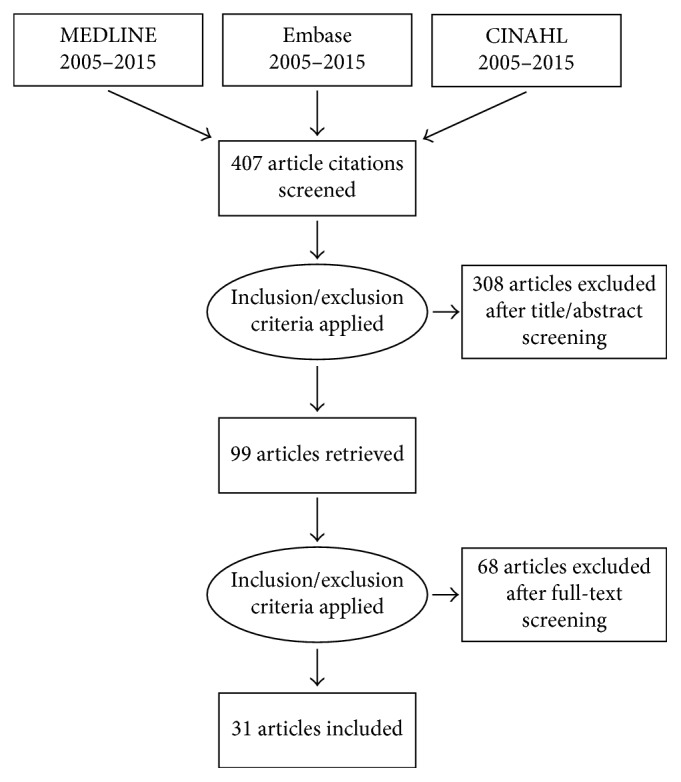
Summary of literature search and included studies.

**Figure 2:**
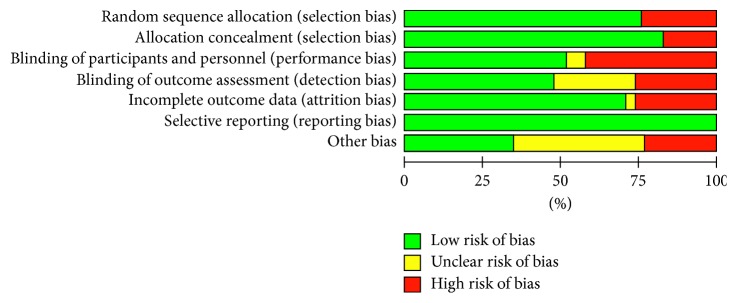
Risk of bias assessment in included studies.

The search identified 407 articles. One author screened the titles and abstracts of these articles, and 308 were excluded. Two authors reviewed the full text of the remaining 99 articles. Of those excluded, 20 were duplicates and 18 were not RCTs. Of the remaining excluded articles, 5 were pediatric studies, 2 were obstetric, 3 were systematic reviews or meta-analyses, 2 were foreign language, 7 did not have postoperative pain as a primary outcome, and 11 were abbreviated studies in supplements so lacked sufficient detail. The results of the literature search are summarised in Figure 1. Thirty-one articles met the inclusion criteria and were included in this systematic review [12–42]. A detailed description of the pain outcomes utilised and timings of measurements in the included studies is provided in Table 1.  Table 2 summarises the number of studies utilizing each pain outcome identified from included studies. Median Jadad score of included studies was 3 (range 2–5). The majority of studies demonstrated a low risk of bias in the 7 domains. A summary of risk of bias assessment is provided in Figure 2.

### 3.1. Postoperative Pain Measurement Tool

Fifteen different outcome measures in total were used in the 31 included studies to assess postoperative pain. The outcome measures utilised in the included studies are summarised in Table 2. The majority of studies (27/31) utilised two or more pain outcomes. The most commonly used outcome measures were numerical grading of pain/numerical reporting scale (NRS) out of 10 (16 studies) [12, 17, 20–24, 27–29, 32–35, 38, 40], opioid consumption (16 studies), and visual analogue scale 10 cm (VAS; 12 studies) [13–16, 25, 26, 31, 36, 37, 39, 41, 42]. Other than reporting total opioid consumption, analgesia usage was also measured with the following outcomes: nonopioid analgesic requirement [14, 16, 32, 33, 36], total supplementary analgesic requirement [34, 35], and cumulative opioid consumption [26, 37]. Other pain outcome measures utilised included: time to first episode of pain [22, 33] and first analgesia or opioid request [18, 28–30, 35–37]. If a study did not use a scoring system to rate pain, the need for analgesia was utilised instead as an outcome measure. There was an evident understanding amongst the selected studies that pain may be worse on movement with separate pain scores (NRS or VAS) taken on activity in 10 of the included studies (Table 2). In the studies that utilised a scoring system to measure pain, there were two groups; those that reported scores at individual time points [12, 14–16, 19, 21, 23–26, 28, 29, 31, 32, 34–37, 39–42] and studies that recorded the worst (or maximum) pain score during the study period [17, 20, 22, 27, 33]. Average pain scores were reported in only one study [38].

### 3.2. Time of Measurement

Time of measurement of pain outcomes also varied with a total of 51 different time points utilised in the 31 studies (Table 1). The time points ranged from immediately following surgery [20, 26, 27, 29, 31, 34, 37, 41] to 12 months postoperatively [25]. Intervals between measurements ranged from every 5 minutes [41] to 6 months [25]. Twenty-two out of 31 of the studies (71%) only evaluated pain over the first 24 hours postoperatively [12, 14, 16, 17, 19, 20, 22–24, 26, 27, 29, 31–34, 36, 38–42].

### 3.3. Nerve Blocks Studied

A variety of nerve blocks were studied (16 in total), which are summarised in Table 3 and may indicate which blocks were seen as important over the study period. Six studies explored continuous infusions [12, 16, 20, 23, 29, 38] with either peripheral nerve or epidural infusions. The remaining studies evaluated single-shot peripheral nerve blocks.

### 3.4. Personnel Collecting Data

Twelve out of the 31 included studies (39%) used an independent or blinded assessor or independent assessment (i.e., postal survey) to assess patients' pain [12, 14, 17, 19, 22, 25–27, 33, 34, 36, 42].

### 3.5. Acute Pain Studies

All but two of the included studies focused on acute pain outcomes. Choi et al. assessed pain outcomes of acute and chronic pain [17] NRS at 4.5 months postoperatively and Wegener et al. [25] looked at the WOMAC (Western Ontario and McMaster Universities Osteoarthritis Index) score and VAS at two different time points (3 months and 12 months).

## 4. Discussion

This systematic review demonstrates that postoperative pain in regional anesthesia RCTs is reported inconsistently. The 31 studies included in this review utilised 15 different types of postoperative pain outcomes, measured at 51 different time points. Therefore at present, there appears to be multiple analyses of different nerve blocks in different centers using different acute pain outcome measures.

Heterogeneity in pain outcomes chosen in the included studies was high. One of the difficulties in deciding which pain outcomes to study in regional anesthesia trials is that there is no reliable method of *objectively* measuring postoperative pain [43]. Physiological parameters, such as heart rate and skin conductance, appear to correlate poorly with pain levels [44, 45]. Instead, pain is often measured by patient-reported intensity, surrogate measures such as the use of supplemental analgesia, or measures of the impact of pain on functioning including the following: sleep, coughing, or ability to perform activities of daily living. Each of these assessment strategies has strengths and limitations, which are demonstrated in this review by the majority of studies using two or more outcome measures to assess pain.

The visual analogue scale (VAS) is a widely used tool to assess postoperative pain. It is considered by some to be more sensitive to fine changes in pain score than numerical scales and four point scales [46]. It also has been shown to demonstrate generally high usability and acceptance; however, elderly patients have been found to not engage with this tool as well as younger patients, since lengthy explanations may be necessary and inconsistent marking along the line has previously been reported [47]. The NRS is another widely used tool to measure pain. Both VAS and NRS are one-dimensional pain tools that are easy to measure and largely reproducible, and thus it may explain why these are often chosen in preference to lengthier multidimensional tools, such as the McGill Pain Questionnaire. Since NRS is a verbal tool, requiring no writing or marking (in contrast to VAS), and is simple to perform by clinical and research team members [43], it should perhaps be considered as an ideal core outcome rather than VAS in studies involving elderly age groups.

Total opioid consumption over the study period (excluding daily and cumulative opioid consumption) was another popular outcome choice, which was utilised in 12 of the 31 studies reporting postoperative pain in this review. This outcome can be interpreted in different ways. A higher total opioid consumption value over a study period is presumed to indicate a higher pain state, necessitating requirement for supplemental opioid-based analgesia. Total opioid consumption could also reflect average pain scores (either NRS or VAS), with higher scores indicating greater opioid requirement. The psychological factors involved in patients requesting additional analgesia warrant further consideration. This involves evaluation of anticipated pain outcome with and without further analgesia, and in order for the request to be made, the patient must feel the treatment of pain outweighs the potential risk of side effects from the drug. This has been shown to be a key decision-making factor when patients are in pain [48]. Total opioid consumption as an outcome may therefore result in patients with different pain states, intensities, and satisfaction levels with analgesia being inappropriately grouped together.

Regional anesthesia is gaining popularity, partly due to improvements in safety and success attributed to ultrasound-guided techniques [49]. The Sprint National Anaesthesia Project (SNAP-1) examined patient-reported outcomes related to satisfaction with anesthesia [50]. Anxiety was found to be the worst part of the perioperative experience. With regard to anesthesia, specific reasons for dissatisfaction: thirst, drowsiness, pain at the surgical site, and hoarseness, were found to be among the most troubling for patients. Regional anesthesia (as a whole) was found to be associated with a reduced burden of side effects. It is unclear what level of pain correlates to adequate patient satisfaction in this population. Nine studies included in this systematic review utilised outcomes consisting of a variant of determinant of effective block duration such as time to first pain or time to first analgesic/opioid request. This suggests that some researchers value the importance of duration of patient being pain-free or experiencing a low enough pain level not to require additional analgesia. However, it should also be noted that a prolonged, dense block may not be in the patients' best interests and may be associated with worse patient satisfaction in this population.

Adequate assessment of pain, using validated tools appropriate to the population or individual, is an essential prerequisite of successful pain management. It has been shown in many countries that inadequate pain assessment is common, with resultant failings in management of pain [51]. Although our review may prove helpful to clinicians and researchers in the future, by summarizing some of the available measures, there are still unanswered questions in this field. In order to assimilate multiple studies with meta-analysis and to derive meaningful clinical conclusions, this review highlights the need for the formulation of a minimum set of outcomes that can be used in future regional anesthesia studies. Use of such a “core set of outcomes” would allow for comparison of outcomes from studies. The COMET or (Core Outcome Measures in Effectiveness Trials) group is a United Kingdom initiative set up in 2010 in response to disjointed outcome measures in clinical research as a whole [52]. Their aim is to standardize outcomes and provide a database from which researchers can access existing outcome sets to design future trials. Specific analysis into the subset of patients undergoing regional anesthesia requires further research. The perspective of patients of the correct demographic (“key stakeholders”) must be considered when deciding core outcomes for postoperative pain assessment in regional anesthesia. This would require exploration of what patients expect following regional anesthesia, including pain expectations following surgery performed with regional anesthesia.

The core outcome set for chronic pain studies may help researchers decide which outcomes to utilize in future regional anesthesia pain studies. A core outcome set of six outcomes for chronic pain was formalised in 2005 by the Initiative on Methods, Measurement, and Pain Assessment in Clinical Trials (IMMPACT) group [53]. This group formalised outcomes to be used for physical functioning, emotional functioning, participant rating for improvement and satisfaction, symptoms and adverse events, and participant disposition, as well as for the assessment of pain. With regards to pain, recommendations included an 11 point 0–10 scale, usage of rescue analgesics, and categorical scale if the patient was unable to use a verbal scale. This systematic review has shown that the IMMPACT recommended pain outcomes for chronic pain are also the most commonly used in the acute pain setting in regional anesthesia RCTs. The 2005 IMMPACT recommendations, which are primarily for improving clinical trial methodology of chronic pain treatments, do not seem to have made any impact on outcomes in regional anesthesia efficacy studies. This may be because the pain outcomes considered clinically important in recovery following elective surgery are different to those important in patients with chronic pain. Acute pain can be reliably assessed, both at rest (important for comfort) and during movement (important for function and risk of postoperative complications), with one-dimensional tools such as NRS or VAS. Chronic pain assessment however and its impact on physical, emotional, and social functions require multidimensional qualitative tools and health-related quality of life instruments [51]. For example, it should be noted that while VAS was found to be one of the most commonly utilised acute pain outcomes identified in this review, it was omitted as an assessment for chronic pain outcomes.

When deciding what should be a “core outcome set,” one must consider if there is an *implied* core set or if there are outcomes that are chosen more commonly among regional anesthesia studies. Until a core outcome set for regional anesthesia pain studies has been formulated, researchers may wish to consider utilizing the most commonly used outcomes identified in this review in order to allow for comparisons between existing data in the literature. It should however be noted that this assumes that the most commonly used outcomes represent what clinicians and researchers believe to be the most important. Based on frequency of utilization, this review suggests that the core outcomes for regional studies exploring acute pain should include NRS (verbal out of 10) at rest, NRS on activity, VAS at rest, total opioid consumption over the study period, analgesic consumption, and time to first analgesic request. The most commonly utilised time points of pain outcome data measurement in the descending order of frequency were 24, 4, 6, 12, and 48 hours postoperatively.

This review does have some limitations. Restricting included RCTs to English language studies may have reduced the number of clinically useful studies analysed. Additionally, the restriction to the top 20 impact factor journals may not reflect the outcome measures utilised in the majority of regional anesthesia studies. This did however serve as a marker of study quality and peer review, which we felt was required in this review. However, there are always risks inherent in limiting groups to be studied. The year of publication of included studies is important to note, and established blocks such as femoral nerve blocks may have already been extensively studied prior to 2005. Use of ultrasound guidance may have made some small differences to pain assessment outcome choice and the debate surrounding adductor canal versus femoral nerve block may continue; however, 2005 to 2017 is a relatively short period of time for major changes in clinical practice to have occurred. We limited the search to articles published over this 12-year period as our intention was to provide the reader with information regarding regional anesthesia studies that would be most relevant to current practice. Finally, although we have attempted to locate all relevant articles by using a robust search methodology, it is possible that with a review of this size, some relevant articles may have been missed. Furthermore, since these studies explore different peripheral nerve and plexus blocks, this may make it more difficult to derive an implied core outcome set from the included group of studies. We appreciate that different surgeries have different temporal pain profiles. Some surgeries for example may peak in pain immediately after surgery, whereas others may have pain that peaks when the nerve block wears off or during days following surgery. However, despite the apparent heterogeneity among the included studies, the vast majority of the RCTs included utilised generic outcomes and only one study used a scoring system specific to the type of surgery performed (the Western Ontario and McMasters Universities Osteoarthritis Index) [25, 54].

In summary, this robust review of the postoperative pain outcomes used in regional anesthesia RCTs between 2005 and 2017 demonstrates significant heterogeneity in choice of outcomes and times of measurements utilised. These findings represent a starting point for further work into developing a core outcome set for future regional anesthesia studies.

## Figures and Tables

**Table 1:** Summary of measurement timings of pain outcomes utilised in included studies.

Author/year	Country of study	Measurement tool	Time measured	Nerve block
Ambrosoli et al. [12]	Not stated	NRS (not stated; 0–10)	4 hours post-op	Sciatic nerve catheter
			Upon discharge	
		NRS at rest (0–10)	24 hours	
			48 hours	
		NRS on activity (0–10)	24 hours	
			48 hours	
		Number of occasions sleep was disturbed by pain	24 hours	
			48 hours	

Andersen et al. [13]	Denmark	Worst pain during knee movement	On day of surgery	Saphenous nerve block
		VAS (10 cm) at rest		
		Time from surgery to VAS score 3 (not stated)	Hours	
		Sleep disturbance due to pain (yes/no)	D1 post-op	
			D2 post-op	
			D3 post-op	
		Total opioid consumption	48 hours	

Bengisun et al. [39]	Turkey	VAS (10 cm; not stated)	2 hours post-op	Interscalene block
			4 hours post-op	
			6 hours post-op	
			12 hours post-op	
			24 hours post-op	

Bharti et al. [14]	India	VAS (10 cm; not stated)	Every 30 min for 2 hours	Supraclavicular brachial plexus block
			Every 1 hour for 6 hours	
			Every 2 hours for 12 hours	
			24 hours post-op	
		Total analgesic requirement (opiate and nonopiate)	24 hours post-op	

Boussofara et al. [15]	Tunisia	VAS (10 cm; not stated)	Every 15 min post-op whilst in PACU	Spinal anaesthetic block
		Total opioid consumption	Whilst in PACU	

Capdevilla et al. [16]	France	VAS (10 cm; not stated)	10 min post-op	Interscalene and popliteal infusions
			1 hour post-op	
			4 hours post-op	
			12 hours post-op	
			AM D1 post-op during physiotx	
			AM D2 post-op during physiotx	
			AM D3 post-op during physiotx	
			AM D4 post-op during physiotx	
		Total analgesic consumption (nonopiate)	Over 72 hours	

Choi et al. [17]	Canada	NRS on activity (0–10)	AM D2 post-op	Femoral nerve block continuous versus single
		Total opioid consumption	48 hours	
		NRS at rest (0–10)	AM D1 post-op	
		NRS on activity (0–10)	D1 post-op	
		Worst NRS (0–10)	D1 post-op	
		NRS (0–10; not stated)	4.5 months post-op	

Diakomi et al. [18]	Greece	Time to first IV opioid request (hours)	No. of hours	Fascia iliaca block
		Total opioid consumption	Over first 24 hours	

Elkassabany et al. [19]	USA	Pain scores (type of pain score not stated)	Before physiotherapy	Femoral nerve block versus adductor canal block
		Pain scores (type of pain score not stated)	After physiotherapy	
		Pain scores (APS-POQ-R)	At 24 hours	
		Total opioid consumption	AM D1 post-op	
			AM D2 post-op	

Fredrickson et al. [20]	New Zealand	NRS (not stated; 0–10)	Emergence	Interscalene catheters
			Worst in 24 hours on movement	
			Worst in 24 hours at rest	
			Worst in second 24 hours on movement	
			Worst in second 24 hours at rest	

Fritsch et al. [21]	Austria	NRS at rest (0–10)	4 hours post-op	Interscalene brachial plexus block
			6 hours post-op	
			8 hours post-op	
			10 hours post-op	
			12 hours post-op	
			14 hours post-op	
		NRS on activity (0–10)	4 hours post-op	
			6 hours post-op	
			8 hours post-op	
			10 hours post-op	
			12 hours post-op	
			14 hours post-op	

Hamdani et al. [38]	Switzerland	Average pain score (NRS; 0–10) (not stated)	Over first 24 hours	Continuous interscalene
		Average pain score (NRS; 0–10) (not stated)	Over first 48 hours	
		Total opioid consumption	Over first 24 hours	
		Total opioid consumption	Over first 48 hours	
		Maximum pain score (NRS; 0–10) (not stated)	Over first 24 hours	
		Maximum pain score (NRS; 0–10) (not stated)	Over first 48 hours	

Karthikeyan et al. [37]	India	VAS (10 cm) (not stated)	Admission to PACU	Bilateral cervical plexus block
			2 hours post-op	
			4 hours post-op	
			6 hours post-op	
			8 hours post-op	
			16 hours post-op	
			24 hours post-op	
		Time to first analgesic request	Min	
		Total analgesic consumption (opioid consumption)	24 hours post-op	

Kim et al. [42]	Republic of Korea	VAS (10 cm; not stated)	1 hour post-op	Serratus-intercostal plane block and intermediate cervical plexus block versus control
			3 hours post-op	
			6 hours post-op	
			9 hours post-op	
			24 hours post-op	

Kulhari et al. [36]	Not stated	Time to first rescue analgesia	After administration of block	Pectoral nerve block versus thoracic paravertebral block
		Total analgesic consumption (opioid consumption)	24 hours post-op	
		VAS (10 cm; not stated)	0 hours post-op	
			0.5 hours post-op	
			1 hour post-op	
			2 hours post-op	
			4 hours post-op	
			6 hours post-op	
			8 hours post-op	
			12 hours post-op	
			24 hours post-op	

Moura et al. [35]	Brazil	NRS (not stated; 0–10)	T0 (after recovering consciousness)	Femoral nerve block
			1 hour post-op	
			2 hours post-op	
		Total dose of supplementary analgesia (opioid and nonopioid)	First 2 hours	
		Time to first analgesic supplementation		

Nagafuchi et al. [34]	Japan	NRS (not stated; 0–10)	Exiting operating room	Femoral nerve block-sciatic nerve block versus femoral nerve block-LIA
			3 hours post-op	
			12 hours post-op	
			24 hours post-op	
		Total dose of diclofenac		

Salviz et al. [33]	USA	Time to first pain	Hours	Interscalene brachial plexus block
		Analgesic consumption (opioid)	D1 post-op	
			D2 post-op	
			D3 post-op	
			D4 post-op	
			D5 post-op	
			D6 post-op	
			D7 post-op	
		Maximum NRS (not stated; 0–10)	D1 post-op	
			D2 post-op	
			D3 post-op	
			D4 post-op	
			D5 post-op	
			D6 post-op	
			D7 post-op	

Sawhney et al. [32]	Canada	NRS on activity (0–10)	D1 post-op	Combined adductor canal block with periarticular infiltration versus adductor canal nerve block
		NRS at rest and with knee bending (0–10)	D1 post-op	
		NRS on activity (0–10)	D2 post-op	
		NRS at rest and with knee bending (0–10)	D2 post-op	
		Analgesic consumption (opioid and nonopioid) per day		

Sato et al. [31]	Japan	VAS (10 cm) at rest	At rest just after surgery	Sciatic and femoral continuous versus single shot
			6 hours after surgery	
			AM D1 post-op	
			PM D1 post-op	
			AM D2 post-op	
			PM D2 post-op	
		Morphine consumption	Over first 48 hours	

Siddiqui et al. [41]	USA	VAS (10 cm) at rest	Every 5 min first hour	Lumbar plexus block
			4 hours post-op	
			8 hours post-op	
			16 hours post-op	
			20 hours post-op	
			24 hours post-op	
			28 hours post-op	
			32 hours post-op	
			36 hours post-op	

Sindjelic et al. [30]	Serbia	Time to first analgesic request	Min	Cervical plexus block
		Total opioid consumption	24 hours post-op	

Schoenmakers et al. [29]	Netherlands	Time to first analgesic request	Min	Popliteal continuous
		NRS at rest (0–10)	Immediately post-op	
			24 hours	
		NRS on activity (0–10)	Immediately post-op	
			24 hours	

Subramanyam et al. [28]	Canada	NRS (not stated; 0–10)	30 min post-op	Supraclavicular brachial plexus block
			60 min post-op	
			90 min post-op	
		Time to first analgesic request	Min	

Stundner et al. [27]	Austria	NRS at rest (0–10)	Baseline before ISB	Interscalene brachial plexus block
			Immediately post-op	
			6 hours post-op worst pain	
			8 hours post-op worst pain	
			10 hours post-op worst pain	
			12 hours post-op worst pain	
			14 hours post-op worst pain	
			AM D1 post-op worst pain	
		NRS on activity (0–10)	Baseline before ISB	
			Immediately post-op	
			6 hours post-op worst pain	
			8 hours post-op worst pain	
			10 hours post-op worst pain	
			12 hours post-op worst pain	
			14 hours post-op worst pain	
			AM D1 post-op worst pain	

Thybo et al. [26]	Denmark	VAS (10 cm) during 30° hip flexion	4 hours post-op (T4) at T0 (pts able to move toes but before SAB worn off)	Lateral cutaneous femoral nerve block
		Pain at rest VAS (10 cm) and during 30° hip flexion	T0	
		Pain at rest VAS (10 cm) and during 30° hip flexion	T1 (after T0)	
		Pain at rest VAS (10 cm) and during 30° hip flexion	T2 (after T0)	
		Pain at rest VAS (10 cm) and during 30° hip flexion	T4 (after T0)	
		Pain at rest VAS (10 cm) and during 30° hip flexion	T8 (after T0)	
		Pain at rest VAS (10 cm) and during 30° hip flexion	T12 (after T0)	
		Pain at rest VAS (10 cm) and during 30° hip flexion	T24 (after T0)	
		Cumulative oxycodone consumption	0–24 hours post-op	

Wegener et al. [25]	Netherlands	WOMAC score	At rest at 3 months	Sciatic nerve block
			On mobilising at 3 months	
			At rest 12 months	
			On mobilising at 12 months	
		VAS (10 cm)	At rest 3 months	
			On mobilising at 3 months	
			At rest 12 months	
			On mobilising at 12 months	
		Oxford knee score (inc. pain)		

Wegener et al. [24]	Netherlands	NRS at rest (0–10)	AM D1 post-op	Sciatic and femoral continuous versus single
			PM D1 post-op	
			AM D2 post-op	
			PM D2 post-op	
			AM D3 post-op	
			PM D3 post-op	
		NRS on mobilisation (0–10)	AM D1 post-op	
			PM D1 post-op	
			AM D2 post-op	
			PM D2 post-op	
			AM D3 post-op	
			PM D3 post-op	
		Total morphine consumption	D0 post-op	
			D1 post-op	
			D2 post-op	
			D3 post-op	

Wongyingsinn et al. [23]	Canada	NRS at rest (0–10)	24 hours post-op	Thoracic epidural block
			48 hours post-op	
			72 hours post-op	
		NRS on walking (0–10)	24 hours post-op	
			48 hours post-op	
			72 hours post-op	
		NRS on coughing (0–10)	24 hours post-op	
			48 hours post-op	
			72 hours post-op	

YaDeau et al. [40]	USA	NRS at rest (0–10)	30 min post-op	Lumbar plexus block
			1 hour post-op	
			2 hours post-op	
			3 hour post-op	
			4 hours post-op	
			24 hours post-op	
		NRS on movement (0–10)	30 min post-op	
			1 hour post-op	
			2 hours post-op	
			3 hour post-op	
			4 hours post-op	
			24 hours post-op	

Zhai et al. [22]	Not stated	NRS at rest (0–10)	Before block	Interscalene brachial plexus block
			Right before discharge from PACU	
			4 hours after block	
			8 hours after block	
			24 hours after block	
		Worst NRS (0–10)	24 hours after block	
		Time of first shoulder pain		

D0 post-op = Day 0 postoperatively; D1 post-op = Day 1 postoperatively; D2 post-op = Day 2 postoperatively; D3 post-op = Day 3 postoperatively; D4 post-op = Day 4 postoperatively; D5 post-op = Day 5 postoperatively; D6 post-op = Day 6 postoperatively; D7 post-op = Day 7 postoperatively; min = minutes; NRS = numeric (verbal) rating scale (0 = no pain to 10 = worst imaginable pain); VAS = visual analogue scale (0 mm = no pain to 100 mm = worst imaginable pain); APS-POQ-R = American Pain Society Patient Outcome Questionnaire Revised; WOMAC score = Western Ontario and McMaster Universities Osteoarthritis Index (5 pain questions included) OA specific; Oxford Knee Score 12-item knee questionnaire on pain; for measurement tool, “not stated” = whether pain score recorded at rest or on movement not stated in methods.

**Table 2:** Summary of pain outcomes reported in included studies.

Pain outcome	No. of studies utilising outcome	Studies
VAS	11	Andersen et al. [13]; Bengisun et al. [39]; Bharti et al. [14]; Boussofara et al. [15]; Capdevilla et al. [16]; Karthikeyan et al. [37]; Kim et al. [42]; Kulhari et al. [36]; Sato et al. [31]; Siddiqui et al. [41]; Wegener et al. [25]

VAS on a specified activity	1	Thybo et al. [26]

Time to VAS 3 cm	1	Andersen et al. [13]

NRS at rest	10	Ambrosoli et al. [12]; Choi et al. [17]; Fritsch et al. [21]; Sawhney et al. [32]; Schoenmakers et al. [29]; Stundner et al. [27]; Wegener et al. [24]; Wongyingsinn et al. [23]; YaDeau et al. [40]; Subramanyam et al. [28]

NRS on activity	9	Ambrosoli et al. [12]; Choi et al. [17]; Fritsch et al. [21]; Sawhney et al. [32]; Schoenmakers et al. [29]; Stundner et al. [27]; Wegener et al. [24]; Wongyingsinn et al. [23]; YaDeau et al. [40]

Maximum NRS score	3	Hamdani et al. [38]; Salviz et al. [33]; Zhai et al. [22]

Average NRS	1	Hamdani et al. [38]

Analgesic consumption	7	Bharti et al. [14]; Capdevilla et al. [16]; Kulhari et al. [36]; Moura et al. [35]; Nagafuchi et al. [34]; Salviz et al. [33]; Sawhney et al. [32]

Opioid consumption	16	Andersen et al. [13]; Bharti et al. [14]; Boussofara et al. [15]; Choi et al. [17]; Diakomi et al. [18]; Elkassabany et al. [19]; Hamdani et al. [38]; Karthikeyan et al. [37]; Kulhari et al. [36]; Moura et al. [35]; Salviz et al. [33]; Sawhney et al. [32]; Sato et al. [31]; Sindjelic et al. [30]; Thybo et al. [26]; Wegener et al. [24]

Time to 1st pain	2	Salviz et al. [33]; Zhai et al. [22]

Time to 1st analgesic request	6	Karthikeyan et al. [37]; Kulhari et al. [36]; Moura et al. [35]; Schoenmakers et al. [29]; Sindjelic et al. [30]; Subramanyam et al. [28]

Time to 1st opioid request	1	Diakomi et al. [18]

Sleep disturbance	2	Ambrosoli et al. [12]; Andersen et al. [13]

WOMAC	1	Wegener et al. [25]

APS-POQ-R	1	Elkassabany et al. [19]

NRS = numerical reported score (verbal; out of 10); VAS = visual analogue scale; APS-POQ-R = American Pain Society Patient Outcome Questionnaire; WOMAC = Western Ontario and McMaster Universities Osteoarthritis Index.

**Table 3:** Summary of regional techniques investigated in included studies.

Blocks studied	Number of studies	Studies
Supraclavicular	2	Subramanyam et al. [28]; Bharti et al. [14]

Interscalene	5	Bengisun et al. [39]; Fritsch et al. [21]; Salviz et al. [33]; Stundner et al. [27]; Zhai et al. [22]

Pectoral	1	Kulhari et al. [36]

Serratus-intercostal	1	Kim et al. [42]

Fascia iliaca	1	Diakomi et al. [18]

Femoral	6	Sato et al. [31]; Wegener et al. [24]; Choi et al. [17]; Thybo et al. [26]; Nagafuchi et al. [34]; Elkassabany et al. [19]

Sciatic	4	Sato et al. [31]; Wegener et al. [25]; Wegener et al. [24]; Moura et al. [35]

Adductor	1	Sawhney et al. [32]

Saphenous	1	Andersen et al. [13]

Cervical plexus	2	Sindjelic et al. [30]; Karthikeyan et al. [37]

Lumbar plexus	2	YaDeau et al. [40]; Siddiqui et al. [41]

Thoracic epidural	1	Wongyingsinn et al. [23]

Spinal	1	Boussofara et al. [15]

Interscalene catheter	3	Hamdani et al. [38]; Capdevilla et al. [16]; Fredrickson et al. [20]

Popliteal catheter	2	Capdevilla et al. [16]; Schoenmakers et al. [29]

Sciatic catheter	1	Ambrosoli et al. [12]

**Table 4:** 

Rank		Impact factor

1	Anesthesiology	5.879
2	Pain	5.213
3	British Journal of Anaesthesia	4.853
4	Pain physician	3.542
5	Anesthesia and Analgesia	3.472
6	Anaesthesia	3.382
7	Regional Anaesthesia and Pain management	3.089
8	Journal of Neurosurgical Anaesthesia	2.99
9	European Journal of Anaesthesia	2.942
10	European Journal of Pain	2.928
11	Canadian Journal of Anesthesia	2.527
12	Clinical Journal of Pain	2.527
13	Pain Practice	2.361
14	Acta Anaesthesia Scandinavia	2.322
15	Minerva Anesthesiology	2.134
16	Journal of Clinical Monitoring and Computing	1.985
17	Current Opinion Anesthesiology	1.979
18	^*∗*^Pediatric Anesthesia	1.85
19	^*∗*^International Journal of Obstetric Anesthesia	1.598
20	Journal of Cardiothoracic and Vascular Anaesthesia	1.463
21	BMC Anesthesiology	1.375
22	Anaesthesia and Intensive Care	1.296

^*∗*^These journals cover obstetric and pediatric anesthesia; hence, they were not used in this study (owing to the inclusion criteria of general adult population).

## Appendix

### A: Literature Search Terms

The basic components of the search were as follows:

  Postoperative pain (ti.ab) AND regional anaesthesia (ti.ab) AND randomised controlled trial (ti.ab) AND top 20 impact factor journals (j.n.) [limited to 2005–2017]

These were the search terms used in OpenAthens to formulate the final search:

1. EMBASE, Medline, CINAHL; (((postoperative pain adj4 pain^*∗*^) OR (post-operative adj4 pain^*∗*^) OR post-operative-pain^*∗*^ OR (post^*∗*^ NEAR pain^*∗*^) OR (postoperative adj4 analgesi^*∗*^) OR (post-operative adj4 analgesi^*∗*^) OR (post-operative adj4 analgesi^*∗*^))) ti.ab
2. EMBASE, Medline, CINAHAL; (((post-surgical adj4 pain^*∗*^) OR (“post-surgical” adj4 pain^*∗*^) OR (post-surgery adj4 pain^*∗*^))) ti.ab
3. EMBASE, Medline, CINAHL; (((“pain-relief after surgery”) OR (“pain following surg”) OR (“pain control after”))) ti.ab
4. EMBASE, Medline, CINAHL; (((“post surg^*∗*^” OR post-surg^*∗*^) AND (pain^*∗*^ OR discomfort))) ti.ab
5. EMBASE, Medline, CINAHL; (((pain^*∗*^ adj4 “after surg$”) OR (pain^*∗*^ adj4 “follow^*∗*^ operat”) OR (pain^*∗*^ adj4 “follow^*∗*^ surg^*∗*^”))) ti.ab
6. EMBASE, Medline, CINAHL; (((analgesi^*∗*^ adj4 “after surg^*∗*^”) OR (analgesi^*∗*^ adj4 “after operat^*∗*^”) OR (analgesi^*∗*^ adj4 “follow^*∗*^ operat^*∗*^”) OR (analgesi^*∗*^ adj4 “follow^*∗*^ surg^*∗*^”))) ti.ab
7. 1 OR 2 OR 3 OR 4 OR 5 OR 6
8. “An?esthesia, Conduction” ti.ab
9. “An?esthesia, Spinal” ti.ab
10. “Analgesi, Epidural” ti.ab
11. “An?esthesia, Epidural” ti.ab
12. “An?esthesia, Caudal” ti.ab
13. “Nerve Block” ti.ab
14. “regional an?esthesia” ti.ab
15. “conduction an?esthesia” ti.ab
16. “spinal block” ti.ab
17. “Epidural block” ti.ab
18. “epidural an?esthesia” ti.ab
19. “plexus block” ti.ab
20. (plexus AND block) ti.ab
21. (bier AND block) ti.ab
22. 8 OR 9 OR 10 OR 11 OR 12 OR 13 OR 14 OR 15 OR 16 OR 17 OR 18 OR 19 OR 20 OR 21
23. (“randomi?ed controlled trial”) OR (“randomi?ed trial”) OR (“controlled trial”) ti.ab
24. EMBASE, Medline, CINAHL; (“anesthesiology” OR “pain” OR “british journal of anaesthesia” OR “pain physician”OR “anesthesia and analgesia” OR “anaesthesia” OR “regional anesthesia and pain medicine” OR “journal of neurosurgical anesthesia” OR “european journal of anaesthesia” OR “european journal of pain” OR “canadian journal of anesthesia” OR “clinical journal of pain” OR “pain practice” OR “acta anaesthesia scandinavia” OR “Minerva anesthesiology” OR “journal of clinical monitoring and computing” OR “current opinion anesthesiology” OR “journal of cardiothoracic and vascular anaesthesia” OR “BMC anesthesiology”).jn
25. 7 AND 22 AND 23 AND 24 [Limit to: Publication Year 2005–2017]

### B: Top Impact Factor Journal List

These are the current impact factors of all the top international anesthesia journals as of 21st October 2015 [55] and were used as limiting functions in the literature review.
